# 96 perfusable blood vessels to study vascular permeability *in vitro*

**DOI:** 10.1038/s41598-017-14716-y

**Published:** 2017-12-22

**Authors:** V. van Duinen, A. van den Heuvel, S. J. Trietsch, H. L. Lanz, J. M van Gils, A. J. van Zonneveld, P. Vulto, T. Hankemeier

**Affiliations:** 1 Division of Analytical Biosciences, LACDR, Leiden University, Leiden, The Netherlands; 2 Mimetas BV, Leiden, The Netherlands; 3 Department of Internal Medicine, Einthoven laboratory for Vascular and Regenerative Medicine, LUMC, Leiden, The Netherlands

## Abstract

Current *in vitro* models to test the barrier function of vasculature are based on flat, two-dimensional monolayers. These monolayers do not have the tubular morphology of vasculature found *in vivo* and lack important environmental cues from the cellular microenvironment, such as interaction with an extracellular matrix (ECM) and exposure to flow. To increase the physiological relevance of *in vitro* models of the vasculature, it is crucial to implement these cues and better mimic the native three-dimensional vascular architecture. We established a robust, high-throughput method to culture endothelial cells as 96 three-dimensional and perfusable microvessels and developed a quantitative, real-time permeability assay to assess their barrier function. Culture conditions were optimized for microvessel formation in 7 days and were viable for over 60 days. The microvessels exhibited a permeability to 20 kDa dextran but not to 150 kDa dextran, which mimics the functionality of vasculature *in vivo*. Also, a dose-dependent effect of VEGF, TNFα and several cytokines confirmed a physiologically relevant response. The throughput and robustness of this method and assay will allow end-users in vascular biology to make the transition from two-dimensional to three-dimensional culture methods to study vasculature.

## Introduction

Disruption of the vascular barrier plays a central role in the onset and progression of diseases, including chronic kidney disease^1^, (vascular) dementia^2,3^, Alzheimer’s^4,5^ and atherosclerosis^6–8^. Preventing the disruption or restoring the barrier is thus an attractive target for drug discovery. However, for in-depth analysis and validation of new drug candidates, we still rely on *in vitro* models that are based on flat monolayers of endothelial cells. These monolayers do not have the complete anatomic architecture of the vasculature, as cells are growing on flat, artificial substrates. Also, important cues in the cellular microenvironment are missing, including interaction with an extracellular matrix (ECM) and exposure to flow. It is crucial to mimic these cues and the three-dimensional morphology found *in vivo* in order to increase the physiological relevance *in vitro* ^9^.

Models that use of microfluidics techniques have recently emerged to increase the physiological relevance *in vitro*, as it allows patterning of cells and hydrogels and ECMs, application of perfusion and spatial control over signaling gradients. Recent reports show microfluidic devices to culture of endothelial cells as perfusable microvessels^10–17^. However, as these devices are early prototypes, we identified three main drawbacks that hinder optimization and adoption of microfluidic methods to culture vasculature^18^. First, there is limited consensus in design and used materials, and many devices are designed from an engineering perspective^19^. This makes direct comparisons between results obtained in these devices difficult, and limits widespread adoption and efficient experimental design. Second, many of silicone-based devices require pre-fabrication before use^10,20^ and require molds (e.g. needles^12,21^ or stamps^10^) to pattern hydrogels. Finally, these devices typically allow for single or low amounts of data points per device and cannot be handled in a high-throughput setting. This hinders optimization of culture conditions and testing of multiple experimental conditions, which renders screening of compounds virtually impossible. As a result, despite the widespread interest in microfluidic cell culture techniques to culture vasculature^22,23^, there are yet no standardized and validated microfluidic assays to study vascular permeability.

Here, we describe a method to culture endothelial cells as 96 individually addressable, three-dimensional microvessels in a standardized microfluidic platform. This platform is based on a microtiter plate format^24^ that was shown compatible with culture of a wide variety of cell types, including neurons^25,26^, intestine^27^ and liver^28^. To mimic the morphology and microenvironment of vasculature *in vivo*, the microvessels are cultured against a 3D scaffold of polymerized collagen-1 and are continuously perfused using a rocker platform. To assess the barrier function, we developed a real-time permeability assay that quantifies the diffusion of 20 kDa and 150 kDa fluorescent dextrans over the vessel wall. This assay was used to optimize the culture conditions for robust and long-term culture of microvessels. Furthermore, we investigated the dose-dependent effect of VEGF, TNFα and several cytokines to study the effect on the permeability of the microvessels.

## Methods

### Cell Culture

HUVEC-VeraVec human endothelial cells (Angiocrine Biosciences, hVera101) were cultured in T75 flasks (Nunc Easyflask, Sigma F7552) with endothelial Cell Growth Medium MV2 (Promocell, C-22022) and used at P3 till P5. Media was replaced three times a week. Cells tested negative for mycoplasma. MV2 endothelial cell Growth Medium (Promocell, C-22022) and Pericyte Growth Medium SR Formulation (Angioproteomie, cAP-09B) were supplemented with 1% pen/strep (Sigma, P4333). M199 medium (Sigma, M4530) was supplemented with 50 µg/mL endothelial cell supplement (Biomedical Technologies, BT-203), 20% Fetal Bovine Serum (Gibco, 16140-071), 1% Antibiotic-Antimycotic solution (Invitrogen, 15240-062), 10 mmol/L HEPES buffer (Invitrogen. 5630-080), 50 µg/mL heparin (Sigma, H3149-100KU) and 2 mmol/L GlutaMAX (Life Technologies, 35050061). All cell culture was performed in a humidified incubator at 37 °C and 5% CO_2_.

The OrganoPlate (Mimetas, 9603-400B) was used for all microfluidic cell culture. Before cell seeding, each observation window was filled with 50 µL HBSS for optical clarity and to prevent gel dehydration. In all experiments collagen type I (R&D systems, 3447-020-01) was used as matrix for the cells to adhere on. A stock solution of 5 mg/mL rat tail collagen type I was neutralized with 10% 37 g/L Na_2_CO_3_ (Sigma, S5761) and 10% 1 M HEPES buffer (Gibco, 15630-056) to obtain a concentration of 4 mg/mL. The neutralized collagen was kept on ice until and used within 30 min. Using a repeater pipette, 2 µL of the neutralized collagen was added into the inlet of each gel channel. To polymerize the collagen, the device was incubated for 30 minutes at 37 °C, 5% CO_2_. After incubation, the device was removed from the incubator and kept sterile at room temperature right before cell loading. Endothelial cells were dissociated, pelleted and resuspended in MV2 medium in a concentration of 2∙10^7^ cells/mL. 2 µL of the cell suspension was dispensed into the perfusion inlet well and the device was placed on its side and incubated for 15 min at 37 °C, 5% CO_2_. After incubation, 25 µL of medium is added in the perfusion inlet well to prevent dehydration of the cell suspension. The plate was placed back in the incubator on its side to allow cells to adhere for at least 45 minutes. After the cells attached to the gel, the plates were rotated back into an upright position and 75 µL of medium was added to the medium outlet. The device was placed on an interval rocker platform for continuous perfusion. (Perfusion rocker, MIMETAS). The rocker was set at a 7 degree inclination and 8 minutes cycle time. Medium was refreshed three times a week and right after each permeability assay.

### Visualization and quantification of permeability

The macromolecular flux of a mixture of two fluorescent labeled dextrans (20 kDa FITC (Sigma, FD20S) and 150 kDa TRITC (Sigma, 48946) was used to quantify and visualize the permeability of the microvessels. The molecular weight of both fluorescence labels is insignificant compared to the molecular weight of the dextrans, thus the fluorescence label does not play a role in the diffusion speed^29^ and the determination of the permeability.

For visualization of the images, the intensities are normalized to the highest intensity in the perfusion channel. To these normalized images, a lookup table is applied to map the color scale, with the highest value of the lookup table set at ¼ of the maximum intensity (0.125 mg/mL). The background intensity (0 mg/mL) is determined by median of the intensity in the gel at t = 0 min and we used this value as lowest value for the lookup table.

For quantification, all images were aligned based on the fluorescent image at t = 0 min. The part containing the microfluidics was cropped and the regions of interest (ROIs) for the perfusion channel and gel channel were manually defined by drawing a ROI for the complete acquired area of the gel channel just below the phaseguide and the ROI of perfusion channel is always selected in the middle along the total length and half the width of the acquired part of the perfusion channel.

The fluorescent intensities of the gel channel and the perfusion channel were quantified using FIJI^30^. The apparent permeability (Papp) in cm/s was derived from formula 1^31^:

1 $${{\rm{P}}}_{{\rm{app}}}(\mathrm{cm}/{\rm{s}})=\frac{dQ}{dt}\cdot \frac{1}{A\cdot {C}_{donor}}$$

where dQ/dt is the flux, A is the surface area in cm^2^ and C_donor_ is the initial fluorescent dextran concentration in the apical side. We assumed a linear relationship between fluorescent intensity and concentration and a starting concentration of zero in the gel. C_donor_ is equal to the intensity in the perfusion channel (I_perfusion_) at the start of the assay. However, as I_perfusion_ changes throughout the experiment due to bleaching or diffusion into the gel, the formula was improved by normalizing the intensity in the gel (I_gel_) to the intensity in the perfusion channel (I_perfusion_)^32^, yielding formula 2:

2 $${P}_{{\rm{app}}}(\mathrm{cm}/{\rm{s}})=\frac{d(\frac{{I}_{gel}}{{I}_{perfusion}})}{dt}\cdot {V}_{gel}\cdot \frac{\,1\,}{A}$$

where I_gel_ /I_perfusion_ is the ratio between the intensity in the gel and perfusion channel, V_gel_ the volume of the gel (4,54∙10^−4^ cm^3^), A the surface area of the microvessel that is in contact with the gel in cm^2^ (1,21∙10^−2^ cm^2^). The ratio (I_gel_/I_perfusion_) was calculated for each individual timepoint. A linear regression was fitted through these ratios to calculate the slope. The slope was multiplied with V_gel_ to obtain the flux over time and divided by A to obtain the apparent permeability.

The apparent permeability includes both the resistance of the cell barrier as well as the resistance in the gel, and the permeability of the microvessel can be derived from the formula:

3 $$\frac{1}{{{\rm{P}}}_{{\rm{app}}}\,}=\frac{1}{{{\rm{P}}}_{{\rm{microvessel}}}\,}+\frac{1}{{{\rm{P}}}_{{\rm{gel}}}\,}$$

where ${{\rm{P}}}_{{\rm{microvessel}}}$ is the permeability of the microvessel and ${{\rm{P}}}_{{\rm{gel}}}$ the permeability of the collagen. However, the diffusion rate of low molecular weight compounds (a hydrodynamic radius below < 8 nm.) in 4 mg/ml collagen does not significantly differ from the diffusion rates in an aqueous solution^33,34^. This suggests that the permeability of the gel $({{\rm{P}}}_{{\rm{gel}}})$ does not significantly contribute to apparent permeability. We verified this experimentally and found that the permeability is around 20 times higher compared to the permeability when a confluent microvessel is present. Thus, we conclude that the influence of the gel can be neglected.

### Media optimization

Both dextrans (25 mg/mL) were mixed and diluted in the appropriate cell culture media to a concentration of 0.5 mg/mL. At the start of the permeability assay, 20 µL of media was added in the gel inlet well, media containing both dextrans was added to both perfusion inlet (40 µL) and the perfusion outlet (30 µL). After addition of the dextran mixture, the device was placed directly inside a conditioned high content imaging system (Molecular Devices, ImageXpress Micro XLS). Fluorescent images were acquired every 3 for 30 minutes. The exposure time was adjusted so the fluorescent intensity in the perfusion channel was below saturation of the detector. After the image acquisition, the plate was removed the media was refreshed to use the plate for additional assays.

### Compound exposure

The microvessels were cultured in MV2 medium for at least 7 days to obtain a proper barrier function. Prior to compound exposure, the microvessels were growth factor starved overnight using basal MV2 supplemented with 0.5% FBS. The compounds used for permeability studies (murine VEGF-165 (PeproTech 450-32), Retinoic Acid (Sigma R2625), Tumor Necrosis Factor α (TNFα) (Sigma T0157), IL8 (ImmunoTools 11349084) and IL1β (ImmunoTools 11343538)) were all aliquoted and stored according to manufacturer’s protocol and diluted in growth factor free MV2 basal medium (Promocell C22221, Germany), supplemented with 0.5% FBS. All compounds were assessed at three concentrations^35^: 1, 10 and 100 ng/mL for IL8, INFγ, RA and TNFα; 10, 100 and 200 ng/mL for VEGF and 0.2, 2 and 20 ng/mL for IL1β. Unless stated otherwise, microvessels were exposed to compounds for 24 hours before doing permeability assays. The permeability assay was performed by diluting 20 kDa FITC- and 150 kDa TRITC-dextran in growth factor free MV2 basal medium to a concentration of 0.5 mg/mL, with 20 µL of media in the gel inlet well, 40 µL of media containing both dextrans to the perfusion inlet and 30 µL of media to the perfusion outlet well. the Fluorescent images were acquired every 3 for 30 minutes, directly after addition of the dextran mixture. After the image acquisition, the plate was removed and media was either refreshed or the microvessels were fixed. Growth factor starved microvessels were refreshed with media containing 5% serum and growth factors to allow them to recover for additional experiments.

### Immunocytofluorescent staining

During all steps of the immunocytofluorescent staining, the device is placed under an angle at all times to create flow, except during staining with primary antibody. Every solution was used in quantities of 100 µL per chip (50 µL in the perfusion inlet, 50 µL in the perfusion outlet) unless specified otherwise. Cells were fixed using freshly prepared 3.7% formaldehyde (Sigma 252549) in PBS. 50 µL of the fixative was added to both the perfusion inlet and outlet for 15 minutes at room temperature (RT), followed by a wash step with 4% FBS in PBS for 5 minutes. After fixation, the cells were permeabilized using 0.3% Trition-X (Sigma T8787) in PBS. After washing, the microvessels were blocked for 45 min using blocking solution (2% FBS, 0.1% Tween20 (Sigma P9169), 2% BSA (Sigma A2153) in PBS). The adherence junctions were visualized using VE-Cadherin (Abcam, 33168, 1:1000 in blocking solution, 30 µL in perfusion inlet, 20 µL perfusion outlet) which was incubated for 1 hr. at RT followed by 30 min. incubation with Alexa Fluor 488 (ThermoFisher Scientific, A11008, 1:250 in blocking solution). To continuously perfuse the chips with primary antibody, the device was placed on a rocker platform. After incubation with the secondary antibody, the device is washed once with washing solution, followed by nuclei staining (NucBlue Fixed cell staining, Life technologies, R37606), and the cytoskeletal marker F-actin staining ActinRed 555 ReadyProbes (ThermoFisher Scientific, R37112) in PBS and imaged using a high content confocal microscope (Molecular Devices, ImageXpress Micro Confocal) at 10x magnification.

### Statistical test

Welch Two Sample t-tests are used to compare treated microvessels with control. Graphs are plotted as mean (SD). Asterisks indicate a significant difference compared to control (P ≤ 0.05). In sample sizes of n > 9, Tukey’s test is used to remove outliers.

## Results

### Microvessel formation and media optimization

**Figure 1:**
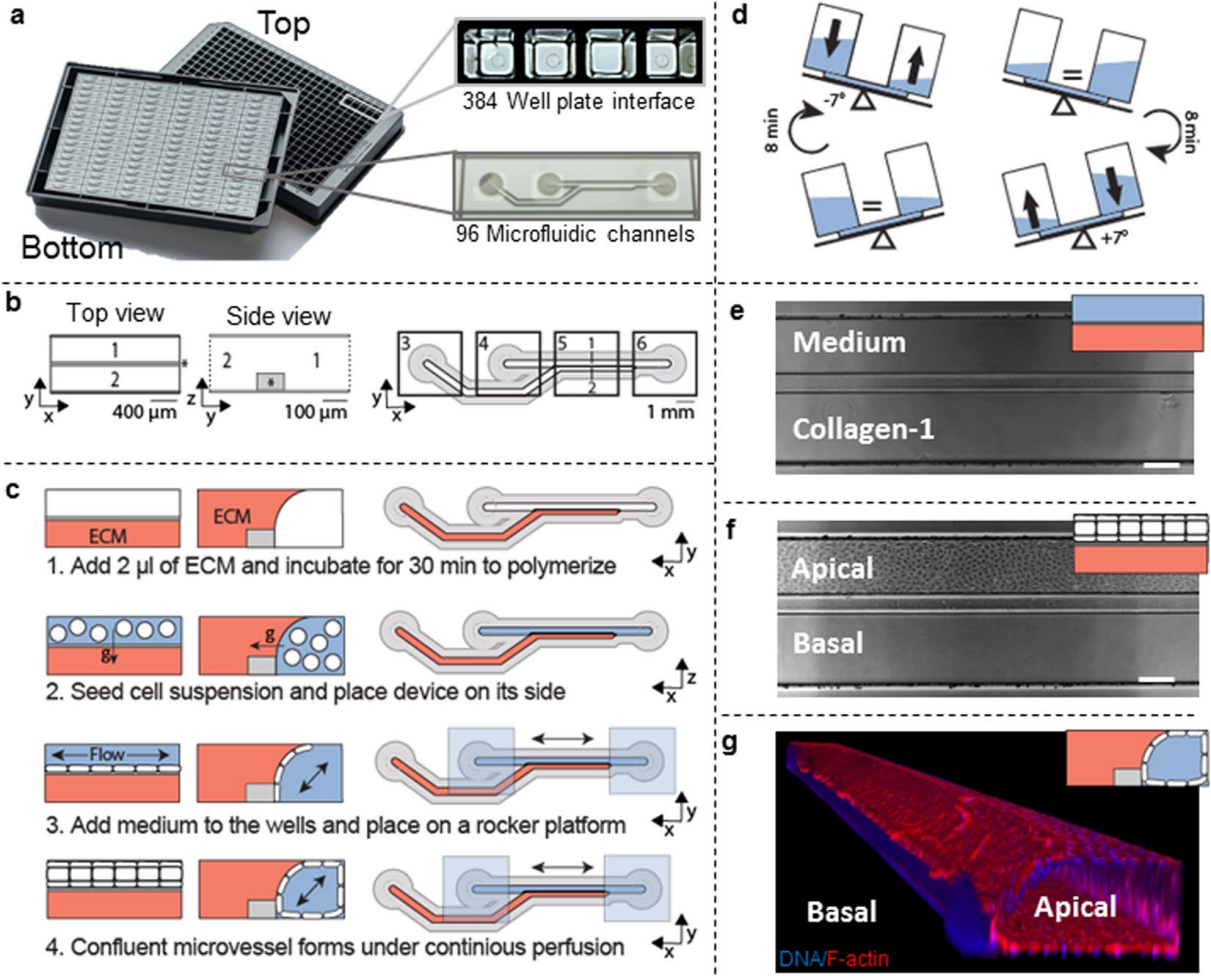
Microfluidic platform for robust culture of perfusable microvessels. (**a**) The microfluidic microtiter plate used for perfusable microvessel culture, based on a 384 wells plate interface on top and 96 microfluidic devices integrated in the bottom. (**b**) Each microfluidic device consists of two channels: an ‘perfusion’ channel (1) and a ‘gel’ channel (2) separated by a phaseguide (*). Every microfluidic structure is positioned underneath 4 adjacent wells. Every first well (3) is positioned on top of the inlet of the gel channel, while every second (4) and fourth well (6) are above respectively the perfusion channel inlet and outlet. Every third well (5) is used for imaging and observation of the experiment. Note that this well does not have an in- or outlet and is therefore not in contact with the microfluidics. Phaseguide, top and bottom substrates are not to scale (**c**) Method for seeding microvasculature. Collagen-1 gel is seeded as extracellular matrix (ECM) and polymerized (step 1). After polymerization, the cells suspension is seeded in the perfusion channel (step 2). The device is placed on its side to allow the cells the settle and adhere to the collagen-1. After adhesion of the cells, perfusion is started by placing the device on a rocker platform (step 3). In 48 hours the cells grow as a confluent monolayer against the collagen gel and channel walls, resulting in a microvessel with a perfusable lumen (step 4). (**d**) The rocker platform creates height differences between the wells, which results in a gravity driven, continuous, bi-directional flow. The device is placed at a 7 degree angle which is inverted every 8 minutes. (**e**) 4× Phase contrast image when imaged below an observation window. Scale bar: 200 µm. (**f**) 48 hr after cell seeding, a confluent vessel of endothelial cells is formed and an apical side (lumen) and basal side (part of microvessel that adheres to collagen-1) can be distinguished. Scale bar: 200 µm (**g**) 3D reconstruction of a DAPI/F-actin stained microvessel.

The platform is based on a standardized microfluidic cell culture platform based on the footprint of a 384 well microtiter plate. It contains 96 microfluidic devices integrated in the bottom, and each single microfluidic device is positioned underneath 4 adjacent wells (Fig. 1a). Each microfluidic device consists of two channels that meet in the center, underneath the third well (Fig. 1b). The channels are separated by a phaseguide, a small ridge that act as a pressure barrier. This enables patterning of cells and the ECM without the use of artificial membranes^36^.

The method to grow microvessels within the microfluidic channels is illustrated in Fig. 1c. First, collagen-1 was seeded into the gel channel (step 1). A droplet of gel on top of the inlet fills the gel channel by capillary force. The phaseguide prevents overflow into the adjacent perfusion channel. After gel loading and polymerization, an endothelial cell suspension was added to the adjacent perfusion channel (step 2). To promote cell adhesion to the collagen-1, the microtiter plate was placed on its side. After the cells adhered to the collagen-1, perfusion was applied by placing the device on a rocker platform (step 3). The rocker platform inverts the angle of inclination (7 degrees) every 8 minutes (Fig. 1d). After 3 days in culture, a confluent microvessel is formed against the collagen-1 (Fig. 1f). When the microvessels are formed, the apical side of the vessel (the lumen) can be accessed through the perfusion channel, while the basal side of the tube is in contact with the collagen-1. Importantly, the presence of a perfusion flow was observed to be crucial for the formation of the microvessels. In the absence of flow, the endothelial cells did not form a confluent monolayer on the bottom of the perfusion channel. Furthermore, after 7 days of culture the microvessels contracted and non-viable cells were visible (Supplementary Fig. 1).

The 3D reconstruction of a microvessel stained for F-Actin and nuclei shows the 3D architecture of the vessel (Fig. 1g) and shows a complete and confluent monolayer and a clear tubular morphology with a perfusable lumen. The curved part adheres to the collagen-1 gel. After 7 days, the morphology of the vessel stabilizes, and can be maintained for at least 60 days. After 60 days, the morphology of the microvessels could not be distinguished from 1-week old cultures. After 60 days, the microvessels were still viable but showed invasion into the adjacent collagen gel (Supplementary Fig. 2).

### Permeability of the microvessels under exposure to different culture media

**Figure 2:**
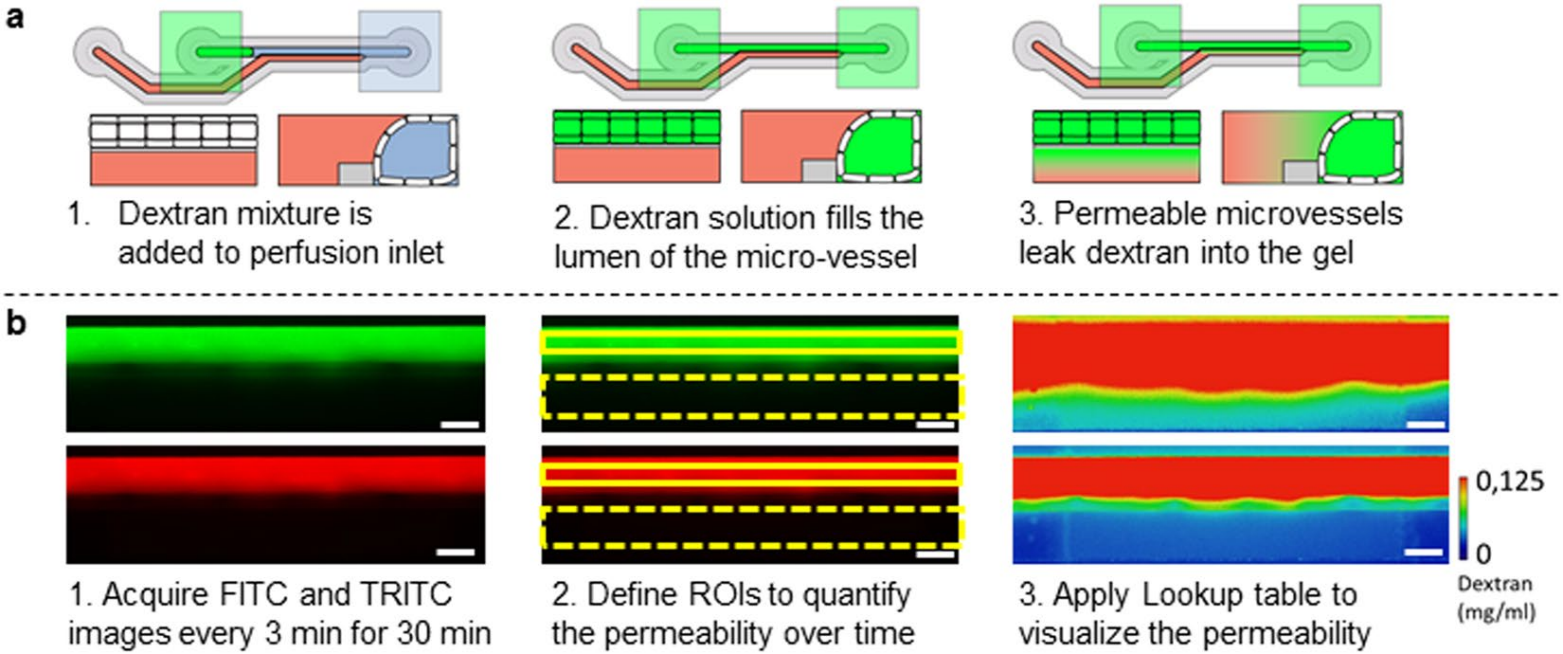
Method for real-time quantification and visualization of the permeability. (**a**) Fluorescent dextran solution is added to the perfusion inlet well. This enters the perfusion channel and completely fills the lumen of the vessel. Without a cells or in case high permeability, the dextran equilibrates between the perfusion and the gel channel. (**b**) For every well, images were acquired every 3 minutes for a total of 30 minutes, resulting in 2112 images per permeability assay. Images are loaded into FIJI and aligned (step 1). Next, the region of interests (ROIs) are defined (step 2) to quantify the permeability to 20 kDa FITC-dextran and 150 kDa TRITC-dextran. For visualization of the permeability, the fluorescent intensity was normalized to the highest value in the perfusion channel and a lookup table was applied to a concentration range between 0 and 0.125 mg/ml dextran. Scale bars: 200 µm.

**Figure 3:**
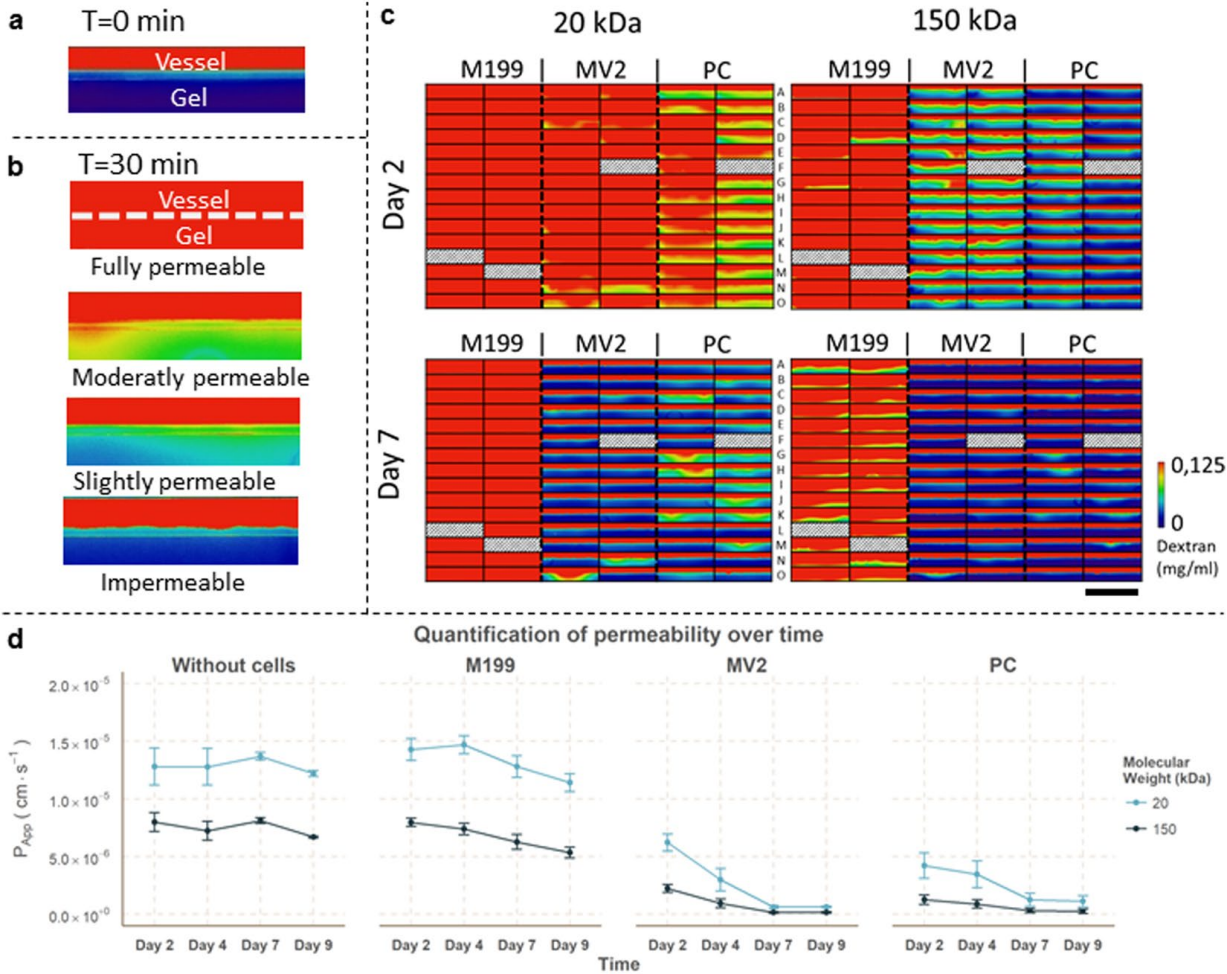
Media optimization on microvessels using the permeability assay. (**a**) At the start of the assay (t = 0 min), the fluorescent dextrans are contained within the microvessel. Scale bar: 200 µm. (**b**) After 30 min, the differences in permeability are clearly visible: the dextran fills the gel in case of a completely permeable microvessel, while an impermeable vessel retains the dextran within the lumen of the vessel. The dotted line indicates the position of the cell barrier. Scale bar: 200 µm. (**c**) Visualization of dextran diffusion after 30 minutes for an array of 86 microvessels with three different culture conditions (M199 medium = M199, MV2 medium = MV2, Pericyte medium = PC). Excluded vessels due improper barrier formation or gel seeding are indicated with a diagonal hatch pattern. Scale bar: 2 mm. (**d**) Quantification of the permeability over different days for M199 (n = 28), MV2 (n = 29) and PC (n = 29). Data is presented as mean ± SD.

Diffusion of dextran into the adjacent collagen gel provides a measure for the permeability of the microvessels. *In vivo*, macromolecules above the molecular weight (MW) of albumin (MW > 70 kDa) are retained in the lumen, while microvessels are permeable to macromolecules smaller then albumin (MW < 70 kDa)^37^. This behavior was simulated by testing the permeability for two different MW dextrans: FITC-Dextran of 20 kDa and TRITC-Dextran of 150 kDa. A mixture of both fluorescent dextrans was added to each perfusion inlet well. This mixture fills into the lumen of the microvessels by passive leveling (Fig. 2a). In the case of a fully impermeable microvessel, the high molecular weight dextran should be retained within the lumen, while for a permeable barrier the dextran diffuses into the gel. Fluorescent images were acquired for 30 minutes with 3 min. interval, yielding a total of 2112 images per permeability assay (Fig. 2b). For quantification, all images were aligned and regions of interest (ROIs) are manually defined. The mean intensity within each ROI is used to calculate the ratio of the fluorescent intensity between the gel and perfusion compartments per time point. The intensity was normalized to the maximum intensity in the perfusion channel and visualized by applying a lookup table (LUT). At the start of the assay (t = 0 min) all dextran is confined to the lumen of the microvessels (Fig. 3a). Differences in permeability are clearly observed at the end of the assay (t = 30 min, Fig. 3b) by the amount of dextran that diffused into the gel.

The permeability of the microvessels was quantified and visualized for three different media compositions: M199, MV2 and Pericyte (PC) medium. After 2 days of culture, 20 kDa dextran diffused into the gel within 30 minutes under all conditions, which indicated that the barrier formation was sub-optimal at this time point (Fig. 3c,d). During the following days, the permeability to 20 kDa and 150 kDa decreased for all media conditions (Fig. 3c,d). Microvessels cultured in MV2 or PC medium are impermeable for 150 kDa dextran after 7 days, while M199 medium still shows leakage of 150 kDa dextran. Importantly, while MV2 and PC grown vessels appear impermeable for 150 kDa dextran, a slight leakage of 20 kDa was still observed. These results suggest that MV2 medium is the optimal choice to study the barrier integrity of the microvessels in this platform and that the microvessels are comparable to microvessels *in vivo*, where microvessels are permeable to compounds with a MW below 70 kDa.

### Effect of VEGF on vascular leakage

**Figure 4:**
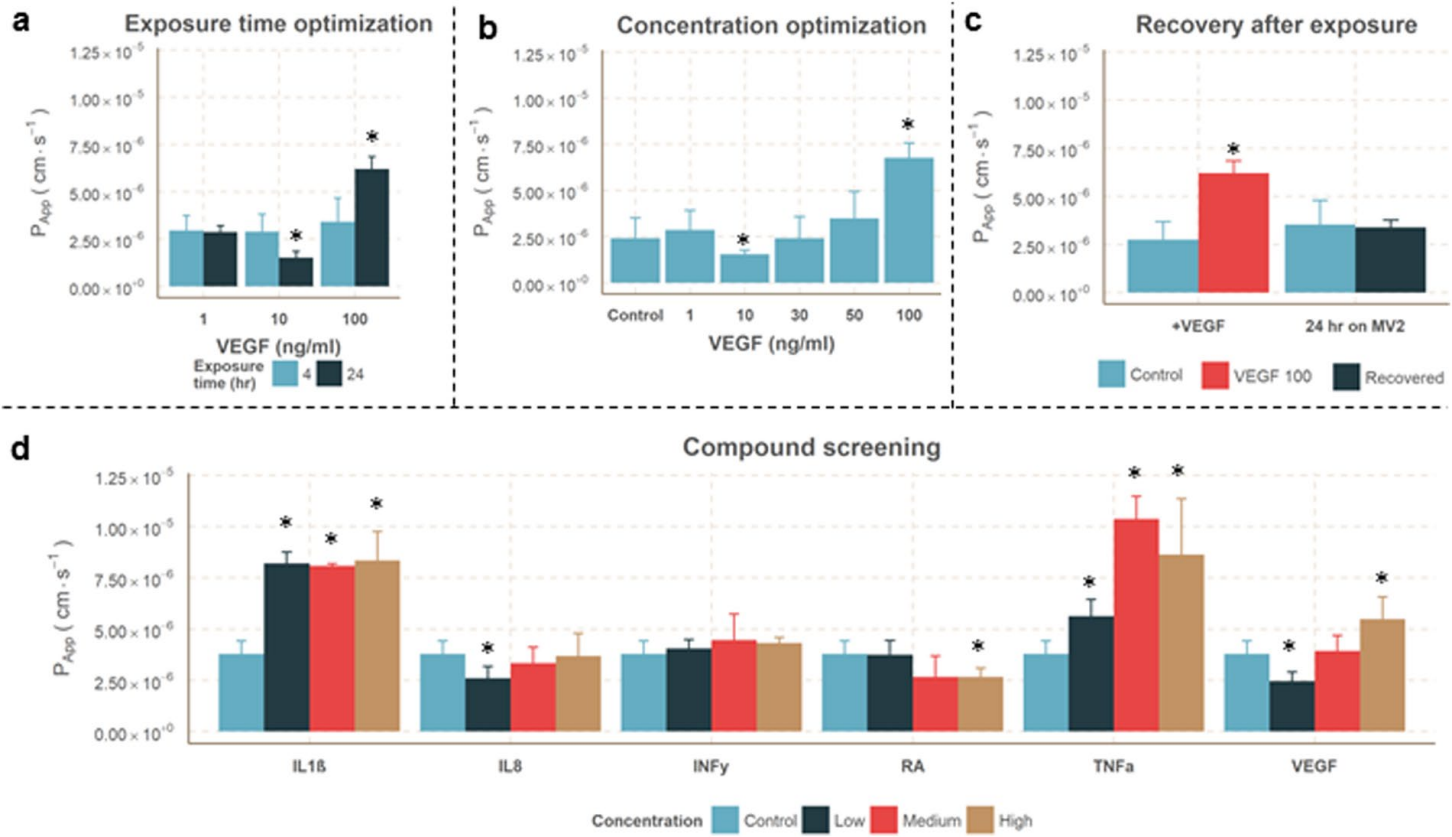
Permeability to 20 kDa dextran after exposures. (**a**) Quantification of the permeability to 20 kDa FITC-dextran after 4 or 24 hr exposure to different VEGF concentrations (n ≥ 5). (**b**) Concentration optimization experiment after 24 hr exposure to VEGF, including 30 ng/ml and 50 ng/ml (Conditions n ≥ 11, gel n = 6). (**c**) Recovery of VEGF stimulated microvessels, where VEGF was removed (n = 5) and replaced with MV2 growth media. 24 hours later the permeability of the recovered microvessels was not significant different to unexposed microvessels (n = 62). (**d**) Permeability after 24 hr exposure to IL1ß, IL8, INFy, RA, TNFα and VEGF in 3 different concentrations. All data is presented as mean ± SD. Asterisks indicate a P-value ≤ 0.05.

Vascular endothelial growth factor (VEGF) is an important and well known modulator of vascular permeability *in vitro* ^38^ and *in vivo* ^39^. We investigated the effect of VEGF on the microvessels after 4 hrs and 24 hrs of exposure. First, the microvessels were cultured for 7 days before removing the growth factors to obtain a proper barrier function. Prior to exposure, the microvessels were growth factor and serum starved overnight. After exposure to VEGF the permeability was quantified by adding the dextran mixture to the lumen of the vessel (Fig. 4a). Although a trend is observed, the permeability that was measured after 4 hours of exposure did not significantly increase. However, after 24 hours the permeability changed significantly. Interestingly, VEGF showed a dose dependent inversion of the effect: 10 ng/mL VEGF showed a significant decrease in permeability compared to control, whereas the highest concentration of 100 ng/mL significantly increased permeability. Another 24-hour exposure including 30 and 50 ng/mL VEGF resulted in a similar response: a significant decrease in permeability at 10 ng/mL VEGF and a significant increase in permeability at 100 ng/mL VEGF (Fig. 4b). To study reversibility of this increased permeability, all media was replaced with MV2 growth media which contained 5% FBS and the complete set of growth factors. It was observed that the permeability of microvessels exposed to 100 ng/mL VEGF returns to control levels after being cultured for 24 hours on MV2 growth media (Fig. 4c). These results show that VEGF modulates the permeability and that this effect is rescued by returning to standard culture conditions.

### Compound screening for induced vascular leakage

Besides VEGF, more cytokines that have a known influence on the endothelial permeability. A range of cytokines which are involved in inflammation have been assessed, including Interleukin 1 beta (IL1β), Interleukin 8 (IL8), Interferon gamma (INFγ), Tumor Necrosis Factor alpha (TNFα). Retinoic acid (RA) was included since it decreases the permeability of endothelium^40^ by upregulating tight junction markers, which are expressed in the blood-brain-barrier^41^. VEGF was added as positive control. All compounds were assessed for three concentrations^35^: 1, 10 and 100 ng/mL for IL8, INFγ, RA and TNFα; 10, 100 and 200 ng/mL for VEGF and 0.2, 2 and 20 ng/mL for IL1β.

IL1β and TNFα treatment resulted in a significantly increased permeability. RA significantly reduced the permeability at high concentrations (Fig. 4d), similar to the effect of 10 ng/mL VEGF. INFγ did not induce a significant change in permeability. These results show that the microvessels have a dose dependent response to cytokines that are known to change the permeability *in vitro* as well as *in vivo*.

**Figure 5:**
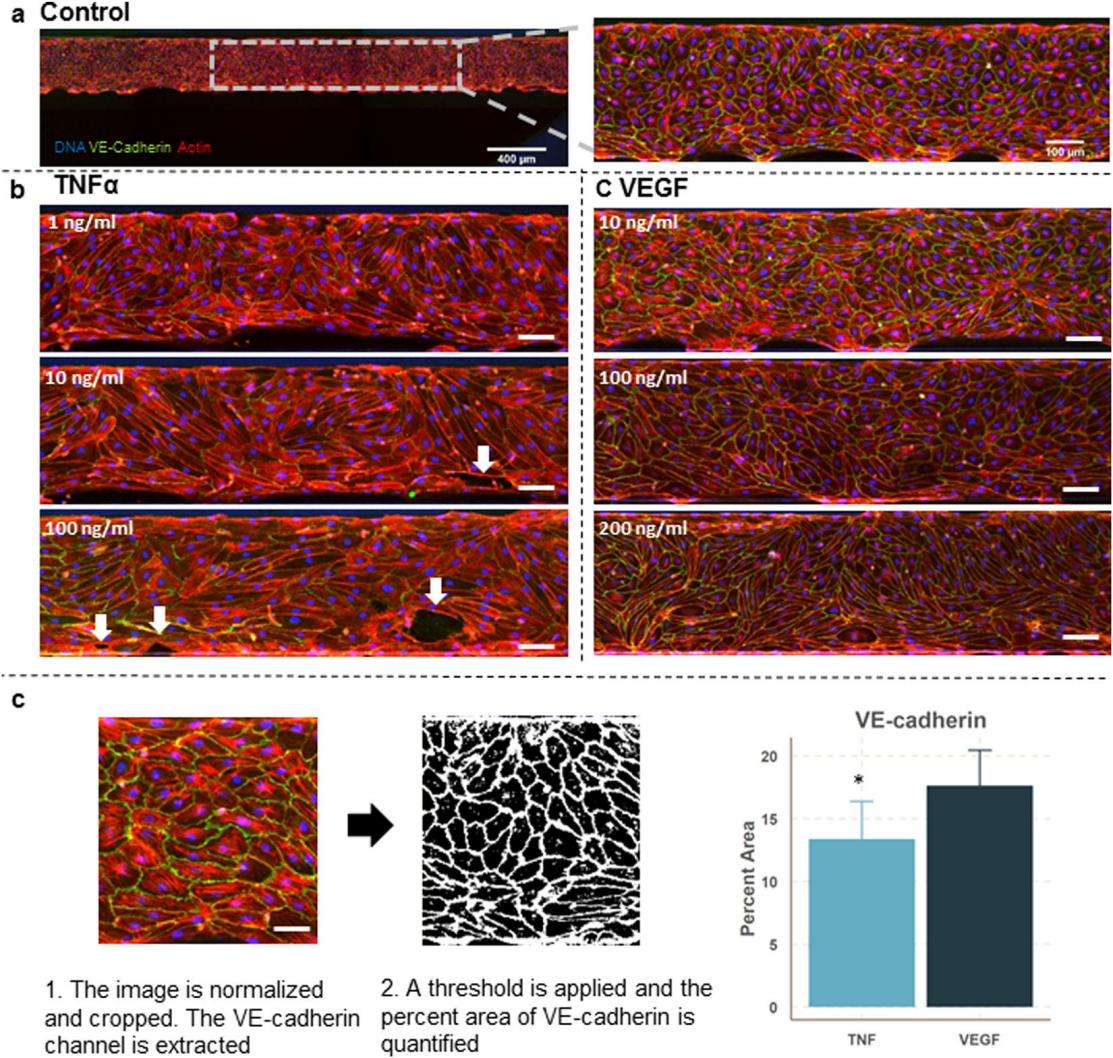
Stained microvessels after 24 hr exposure to TNFa or VEGF. (**a**) The microvessels are stained for VE-cadherin (green), F-actin (red) and the nuclei (blue) and the bottom of every microvessel was imaged using confocal microscopy. Image on the left is a stitch which consists of three images. The picture on the right is a close-up to highlight the typical cobblestone appearance with uniform expression of VE-cadherin around the cell borders. (**b**) Stained microvessels treated with different concentrations of TNFα. Shown images are representative of 3 microvessels per condition. All microvessels show a decreased expression of VE-cadherin and induction of actin stress fibers compared to control. Arrows indicate the large perforations in the microvessels. Scale bars are 100 µm. (**c**) Stained microvessels treated with different concentrations of VEGF. After 24 hour exposure to 10 ng/ml VEGF, the microvessels show VE-cadherin expression which is comparable to control levels. In contrast, 100 ng/ml and 200 ng/ml VEGF induced elongation and stretching of the cells, while VE-cadherin expression is comparable to control levels. Shown images are representative of 3 microvessels per condition. Scale bar is 100 µm. (**d**) Difference in VE-Cadherin between TNFα exposed and VEGF exposed microvessels. Data is presented as mean ± SD.

The microvessels exposed to TNFα and VEGF were fixed and stained for DNA (Hoechst), VE-cadherin (antibody) and F-actin (phalloidin) (Fig. 5). The vessels treated with TNFα show a decreased expression of VE-cadherin and decreased alignment of the actin fibers. At the two highest doses, large perforations in the monolayer could be distinguished, which correlates nicely with the permeability values. TNFα-treated microvessels show actin stress fibers in the direction of the elongated cell axis in combination with a reduction of VE-cadherin expression. In contrast, microvessels exposed to VEGF do show an increased permeability, but the expression of VE-cadherin is not affected. This shows that in this platform, the permeability assay and immunocytofluorescent staining can be easily combined to get a more comprehensive overview of the mechanism of action.

## Discussion

Microfluidics has a clear potential to add physiological relevant cues to vasculature *in vitro* (e.g. perfusion, adherence to an ECM)^22,42^, and there are various examples of the culture of 3D, perfusable microvessels in microfluidic platforms. However, unlike other microfluidic platforms which demonstrate platforms with a few replicates per device, this is the first platform that has the required throughput to be used as an robust screening assay. Furthermore, it is usable in a general cell culture laboratory, as the microtiter format is compatible with almost all laboratory equipment, including multichannel pipettes and automated microscopes. Also, since flow is induced by passive leveling instead of pumps, contamination and handling issues are minimized, while scalability and throughput is ensured. The microvessels can be maintained over prolonged periods of time and a robust assay has been developed to quantify the permeability in real-time.

Compared to traditional 2D-based assays, this platform has a comparable throughput (n = 96) while it has several advantages. First, 2D-based macromolecular diffusion assays are based on horizontally stacked membranes, which limits the possibility to image leakage in real-time. In contrast, the permeability assay presented here allows correlating real-time permeability with phenotypic screening, and the permeability can be determined multiple times over the course of days or weeks. This can reveal interesting differences, as for example morphologically identical microvessels show a different permeability (Supplementary Fig. 2). Also, immunocytofluorescent stainings can be easily combined with the permeability assay to get a more comprehensive insight into the mechanisms behind induced permeability, which adds a valuable tool to the high-content imaging toolbox. Another advantage over 2D-based assays is the possibility of patterning of gels and ECMs. By providing the cells a soft matrix, the gene expression and morphology comes closer to of that *in vivo* ^9^.

The robustness and throughput of this method is clearly illustrated in Fig. 3. In principle, 96 microvessels can be formed that have a significant barrier in 7 days. The different culture media clearly show a difference in barrier function of the microvessels. The serum concentration in each culture media is different. This might explain the observed differences, as serum contains factors that module the permeability. Under optimal culture conditions, our results show that the microvessels have a size-selective permeability: 150 kDa TRITC-Dextran is confined to the lumen, while 20 kDa FITC-Dextran diffused into the interstitial space. The permeability value we observed are comparable to vasculature *in vivo* ^37^. Furthermore, it is shown that microvessels *in vivo* have shown a size selective permeability^43^. Although a range of literature reports have created perfused vasculature in microfluidic culture platforms prior to this publication only very few have convincingly demonstrated impermeability to high molecular weight compounds^11^ and none of them have shown this throughput and robustness over time.

Inducing perfusion by passive leveling results in a bidirectional, oscillating flow, and the microvessels is this platform are periodically exposed to significant levels of shear (5 dyne/cm^2^) right after the rocker position has been changed. We did not observe alignment of the endothelial cells to the flow direction, which could be due the absence of continuous, unidirectional flow. *In vivo*, similar-sized venules are exposed shear levels between 1 to 5 dyne/cm^2^. It is shown *in vitro* that exposure to unidirectional flow in combination with high levels of shear (7–10 dyne/cm^2^)^44,45^ decreases the permeability of endothelial monolayers. Nonetheless, our experiment show that the microvessels are still able to form a significant barrier function with bi-directional flow and lower shear. We aim to compare the effect between bidirectional and unidirectional flow with various levels and durations of shear stress on the permeability of the microvessels.

In our experiments, collagen type I was used as matrix for cells to adhere on. Interestingly, it is shown that collagen type I and IV promote angiogenesis and tube formation in other *in vitro* platforms, while laminins stabilize the endothelium^46^. However, the microvessels in this platform did not show any invasive behavior or angiogenic sprouting into the collagen. This suggests that the endothelial cells are in a more quiescent state than a proliferative, invasive state. However, we have shown that when stimulated with right combination of angiogenic factors on the basal side, the microvessels are able to form angiogenic sprouts into the collagen-I gel^47^. As this platform allows the integration of different ECMs or ECM-derived components (e.g. laminins or different types of collagen), it will be a valuable tool to decipher the role of ECM and proteins in the stabilization and maturation of vasculature.

The inflammatory cytokines TNFα and IL1β both significantly increased the permeability of our microvessels, an effect that is extensively described *in vitro* ^35,48–50^ and *in vivo* ^51,52^. INFγ exposure did not result in a significant change in permeability, which is also shown in impedance assays^35^. The response to VEGF is different from other *in vitro* assays. In traditional membrane based studies, VEGF linearly increases the permeability of the monolayer^38,49^, but our results show a biphasic response to VEGF exposure. The barrier protective effect of 10 ng/mL VEGF is shown by one other study, were it is contributed to the induction of cAMP^53^. cAMP is linked to barrier protective properties and explains the reduction in permeability^54^. Future studies will include the characterization of the effect of multiple cytokines or the combination of cytokines with inhibitors to elucidate the most important pathways that induce or prevent a change in permeability in this model.

A standardized platform like the one presented here will contribute to the transition from static, membrane-based, 2D culture techniques to more physiological relevant 3D culture methods. This will increase the efficiency of pre-clinical testing and validation of new lead compounds. In future work, the flexibility of the platform can be further leveraged by including other relevant cell types found in the vascular microenvironment. This allows the study of for example the detachment of pericytes from the vasculature, which is an important hallmark in for example rarefaction^1^. A more physiologically relevant *in vitro* model of vasculature will help to elucidate the key mechanisms behind the onset and progression of these and other diseases.

## Conclusion

We developed and optimized a robust, high-throughput permeability assay to assess the barrier integrity of three dimensional, perfusable microvessels in high numbers (up to 96 per plate) against a collagen-1 matrix. We have shown that the microvessels have a size selective permeability which can be correlated with *in vivo* data. The permeability is influenced after exposure to cytokines which are involved in inflammation. The throughput and compatibility of the platform as well as the availability of tailored assays make the platform ready for adoption by end-users in vascular biology.

## Electronic supplementary material

Supplementary info
