# Supplementary material for: 96 perfusable blood vessels to study vascular permeability in vitro
Source: Sci Rep. 2017 Dec 22;7:18071. doi: 10.1038/s41598-017-14716-y (PMC5741747; doi:10.1038/s41598-017-14716-y)
Supplement: Supplementary file 1 — Supplementary info [file 41598_2017_14716_MOESM1_ESM.docx]

**Supplementary information 96 perfusable blood vessels to study vascular permeability *in vitro***

V. van Duinen^1^, A. van den Heuvel^2^, S.J. Trietsch^2^, H.L. Lanz^2^, J.M van Gils^3^, A.J. van Zonneveld^3^, P. Vulto^2^, T. Hankemeier^1*^

1. Division of Analytical Biosciences, LACDR, Leiden University, The Netherlands, 2. Mimetas BV, Leiden, The Netherlands, 3. Department of Internal Medicine, Einthoven laboratory for Vascular and Regenerative Medicine, LUMC, Leiden, The Netherlands

**
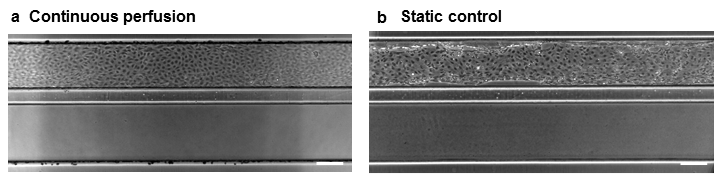
Supplementary Fig 1. Culture of microvessels with and without perfusion after 7 days**

A) Microvessels cultured for 7 days on rocker platform grow as a confluent monolayer against all surfaces. No contraction is observed and all cells are viable. B) Without perfusion, microvessels shows severe contraction after 7 days of culture and non-viable cells are visible. Scale bars: 200 µm.

**
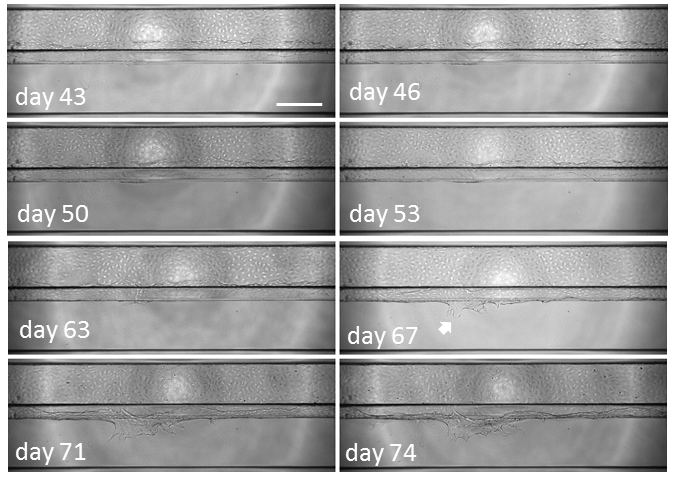
**

**Supplementary Fig 2. Prolonged culture of microvessels.**

Microvessels show a stable morphology for up to around 60 days. At day 67, cells start to invade the collagen matrix and 2D outgrowth is visible (arrowhead). Scale bar: 400 µm.

**
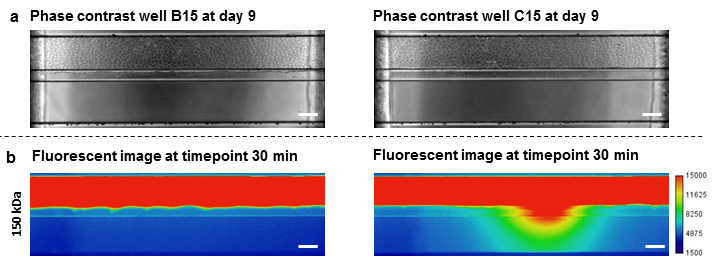
**

**Supplementary Fig 3.: Morphological similar tubes show differences in permeability.**

A) Two microvessels after 9 days of culture on MV2 medium, which appear to be identical when imaged with phase contrast. B) The same microvessels show clear differences in permeability when performing a permeability assay. While the left vessel is impermeable for 150 kDa, the right microvessel is permeable for 150 kDa dextran and a major point of permeability can be identified. Scale bars: 200 µm.
